# Can Ginseng Alleviate Cancer-Related Fatigue?

**DOI:** 10.6004/jadpro.2013.4.6.1

**Published:** 2013-11-01

**Authors:** Pamela Hallquist Viale

As many of you have probably noticed, JADPRO’s review series for 2013 has focused on topics related to integrative medicine. We’ve covered hypnosis as a modality for selected patients with cancer, the use of touch and energy healing therapies, and complementary strategies for the side effects of radiation therapy, among others. Although the use of integrative therapies and natural agents has not been adopted in all hematology and oncology settings, the study of these intriguing treatments continues. One thing is clear: Further rigorous research is needed to determine the safety and therapeutic value of these interventions in our patient population.

## Ginseng and Fatigue

The healing properties of one natural agent in particular, ginseng, have been touted for millennia. One recently published study does much to validate the use of ginseng in patients with a particularly devastating symptom: cancer-related fatigue. Barton and colleagues published their results of the use of Wisconsin ginseng in a large trial of 364 patients from 40 different institutions (Barton et al., 2013). Their findings showed positive results in combating this common yet often debilitating symptom.

Fatigue, which affects over 60% to 90% of patients with cancer, is usually more severe as the disease progresses (Lawrence, Kupelnick, Miller, Devine, & Lau, 2004). The National Comprehensive Cancer Network describes cancer-related fatigue as distressing and persistent; exhaustion related to cancer or cancer treatment interferes with usual functioning (Piper & Cella, 2010). In one retrospective study of 1,778 patients, 84% presented with moderate to severe fatigue, with pain and appetite changes being the most significant predictors of this symptom (Yennu, Urbauer, & Bruera, 2012).

Barton and colleagues reported on their multisite trial (N07C2) in the *Journal of the National Cancer Institute* (2013). The study was a double-blind one in which 364 fatigued cancer survivors were randomized to receive 2,000 mg of American ginseng vs. a placebo for a total of 8 weeks. The primary endpoint of the study was the 4-week measurement of fatigue on the general subscale of the Multidimensional Fatigue Symptom Inventory-Short Form (MFSI-SF), with changes noted from baseline at 4 and 8 weeks and evaluated by a two-sided, two-sample t-test.

## Intriguing Study Results

The results reported by Barton and colleagues showed that changes in MFSI-SF score from baseline to 4 weeks were 14.4 (standard deviation [SD] = 27.1) for the patients receiving ginseng vs. placebo (8.2 with SD = 24.8;* p* = .07). At 8 weeks, a statistically significant difference was also seen for the ginseng group, with a change score of 20 (SD = 27), whereas the placebo group scored 10.3 (SD = 26.1;* p* = .003). Patients who were receiving active therapy for their disease had better scores than the patients who had already completed therapy. Toxicities were similar between groups.

## What Does Ginseng Do?

Several types of ginseng exist, although the type most commonly used in herbal preparations is Asian ginseng. The study discussed here used American ginseng. Ginseng has been reported to have many different effects, including sedation, antidepressant activity, diuresis, and aphrodisiac properties (Sparreboom, Cox, Acharya, & Figg, 2004). Reports of research showing a dose-response relationship between ginseng and a decrease in cancer have been published (Yun & Choi, 1998).

The active constituent in extracts of ginseng is ginsenoside, which could possibly cause the drug interactions that may occur with the use of this product (Sparreboom et al., 2004). Since the toxicities seen in the N07C2 trial did not differ statistically between the two arms, it may be presumed that significant drug-drug interactions did not occur in this study; this is an important fact, as adverse events could be problematic if the compound interfered with or caused additional side effects.

As the results support using this treatment for cancer-related fatigue, oncology advanced practitioners could consider ginseng therapy an option for their patients experiencing fatigue. However, continued research is needed to definitively study the role of ginseng in the treatment of cancer-related fatigue, a symptom that is prevalent and devastating to so many of our patients. The Barton et al. study represents an important addition to that body of research.

## JADPRO Live!

If you haven’t already signed up to attend JADPRO Live, our first live educational symposia to be held in sunny St. Petersburg, Florida, from January 24-26, 2014, you still have time to do so! The meeting will target advanced practitioners working in oncology and hematology and will focus on making the most of collaborative practice. Our renowned faculty includes physicians, advanced practice nurses, physician assistants, and other recognized professionals in the field. Updates in the treatment of various cancers will be provided and participants will have the opportunity to discuss, network, and learn about critical issues in the care of our oncology patients today.

Our conference will open with a panel discussion on leadership perspectives regarding the role of the advanced practitioner in oncology care today with Dr. Steven Allen of ASH, Dr. Robert Carlson of the NCCN, Dr. Louis Harrison of ASTRO, and Dr. Peter Yu of ASCO as discussants. Laura Zitella, NP, will moderate a special panel regarding the advanced practice horizon in the constantly changing environment of health care in 2014 and beyond. Don’t miss this unique two-day meeting where your issues will be heard. Come see us in Florida!
